# Low GRK2 Underlies Hyperalgesic Priming by Glial Cell-Derived Neurotrophic Factor

**DOI:** 10.3389/fphar.2018.00592

**Published:** 2018-06-05

**Authors:** Hui-Jing Wang, Han-Xin Gu, Niels Eijkelkamp, Cobi J. Heijnen, Annemieke Kavelaars

**Affiliations:** ^1^Laboratory of Neuropsychopharmacology, College of Fundamental Medicine, Shanghai University of Medicine & Health Science, Shanghai, China; ^2^Laboratory of Neuroimmunology and Developmental Origins of Disease, University Medical Center Utrecht, Utrecht, Netherlands; ^3^Division of Internal Medicine, Laboratory of Neuroimmunology, Department of Symptom Research, The University of Texas MD Anderson Cancer Center, Houston, TX, United States

**Keywords:** GDNF, hyperalgesic priming, GRK2, Epac, HSV vector, ESI-09

## Abstract

**Background:** We recently identified the balance between the level of G protein coupled receptor kinase 2 (GRK2) and Epac1 in nociceptors as a key factor in the transition from acute to chronic pain that occurs in mice ‘primed’ by an inflammatory stimulus. Here, we examined the contribution of GRK2 and Epac-signaling to growth factor-induced hyperalgesic priming.

**Methods:** Mice were primed by intraplantar injection with glial cell-derived neurotrophic factor (GDNF). Mechanical allodynia in response to PGE_2_ was followed over time in primed and non-primed animals. GRK2 protein levels in dorsal root ganglion (DRG) neurons were quantified by immunohistochemistry. The effect of herpes simplex virus (HSV)-GRK2 amplicons to restore GRK2 levels or of an Epac inhibitor on PGE_2_ allodynia in primed mice was examined.

**Results:** Glial cell-derived neurotrophic factor-induced hyperalgesia disappeared within 12 days. The hyperalgesic response to a subsequent intraplantar injection of PGE_2_ was prolonged from <24 h in control mice to more than 72 h in GDNF-primed mice. In male and female primed mice, PGE_2_ hyperalgesia was inhibited by oral administration of the Epac inhibitor ESI-09, while the drug had no effect in control mice. Mice primed with GDNF had reduced levels of GRK2 in IB4(+) small DRG neurons, but normal GRK2 levels in IB4(-) DRG neurons. Intraplantar administration of HSV-GRK2 amplicons to increase GRK2 protein levels prevented the prolongation of PGE_2_-induced hyperalgesia in GDNF-primed mice.

**Conclusion:** Low GRK2 in nociceptors is critical to develop a primed state in response to GDNF and leads to engagement of Epac signaling and transition to chronic PGE_2_-induced hyperalgesia. Increasing GRK2 protein or inhibiting Epac signaling may represent new avenues for preventing transition to a chronic pain state.

## Introduction

The mechanisms underlying the development of persistent pain are poorly understood and this limits identification of novel therapeutic strategies to combat chronic pain. The animal model of hyperalgesic priming has been developed as a tool to study the transition from acute to chronic pain (Aley et al., 2000; Joseph et al., 2003; Reichling and Levine, 2009; Asiedu et al., 2011; Kim et al., 2016). In our previous studies, we showed that hyperalgesic priming in response to the inflammatory stimulus carrageenan is caused by a persistent reduction in the level of G protein-coupled receptor kinase 2 (GRK2) in nociceptors (Wang et al., 2013). GRK2 can specifically recognize and desensitize agonist-activated G protein-coupled receptors (GPCRs) by phosphorylating intracellular serine and threonine residues leading to uncoupling from the G protein and arrestin-mediated internalization of the receptor (Iyer et al., 2006; Singhmar et al., 2016). Besides regulating cellular signaling at the level of GPCRs, GRK2 also directly interacts with intracellular signaling molecules, including the exchange protein activated by cAMP (Epac), extracellular signal regulated kinase 1/2, phosphoinositide-3 kinase and p38 mitogen-activated protein kinase (Naga Prasad et al., 2002; Liu et al., 2005; Jimenez-Sainz et al., 2006; Peregrin et al., 2006; Eijkelkamp et al., 2010b; Singhmar et al., 2016).

By using mice with global or cell-specific partial deletion of GRK2, we have demonstrated that the intracellular level of GRK2 determines the duration of inflammatory hyperalgesia. Mice with a global ∼50% reduction in GRK2 develop prolonged and/or increased pain behavior in response to carrageenan, PGE_2_, epinephrine, IL-1β, and CCL3 (Eijkelkamp et al., 2010b; Willemen et al., 2010; Wang et al., 2011). Moreover, we demonstrated that a reduction in nociceptor GRK2 protein level was sufficient to prolong pain induced by PGE_2_ and epinephrine (Eijkelkamp et al., 2010a; Wang et al., 2011).

In naïve animals PGE_2_ and epinephrine activate the cAMP/PKA pathway, leading to transient hyperalgesia (Villarreal et al., 2009; Eijkelkamp et al., 2010b; Ferrari et al., 2015). However, in mice heterozygous for deletion of GRK2 in Na_V_1.8^+^ neurons, PGE_2_-induced allodynia is no longer dependent on PKA but is routed via cAMP signaling to Epac, leading to prolonged allodynia (Kawasaki et al., 1998; Eijkelkamp et al., 2010b; Gloerich and Bos, 2010; Singhmar et al., 2016). Epacs are guanine nucleotide exchange factors (GEFs) catalyzing the exchange of GDP for GTP for the Ras-like GTPases Rap1 and Rap2, resulting in their activation. We showed recently that phosphorylation of Epac1 by GRK2 inhibits the Epac1 mediated activation of Rap1 (Eijkelkamp et al., 2010b; Wang et al., 2011, 2013) and thereby prevents chronic pain. In addition, we showed that the Epac inhibitor ESI-09 can inhibit chronic pain (Villarreal et al., 2009).

In the carrageenan model of hyperalgesic priming in female mice, we showed that normalization of nociceptor GRK2 in primed mice by viral-based gene transfer or reducing Epac1 by intrathecal administration of anti-sense oligonucleotides prevented the prolongation of PGE_2_-induced allodynia (Wang et al., 2013).

It has been reported that GDNF sensitizes IB4(+) afferent nociceptor populations (Amaya et al., 2004; Malin et al., 2006; Bogen et al., 2008), producing transient mechanical hyperalgesia and a prolonged pain response to subsequent stimulation with PGE_2_ (Alvarez et al., 2012; Hendrich et al., 2012). Furthermore, by using the model of priming, Levine colleagues have demonstrated that GDNF-induced hyperalgesic priming is restricted to IB4(+) nociceptors and depends on PKCε signaling pathway (Joseph and Levine, 2010). However, whether our previous findings that GRK2 and Epac1 are important in inflammatory hyperalgesic priming (Wang et al., 2013) are also applicable to GDNF-induced priming is not known. Moreover, the results of GDNF-induced priming were achieved in male rats, while our previous studies on the role of GRK2 and Epac in priming were performed in female mice. Here, we addressed the question whether the role of GRK2 and Epac signaling in hyperalgesic priming extends to the GDNF model of hyperalgesic priming in both male and female mice.

## Materials and Methods

### Animals

Female and male C57Bl/6 mice aged 12–14 weeks were used. Mice were maintained in the animal facility of the University of Utrecht (Netherlands) and the animal facility of the Shanghai University of Medicine & Health Science (SUMHS). All experiments were performed in accordance with international guidelines and approved by the University Medical Center Utrecht experimental animal committee and the animal ethics committee of SUMHS. All experiments were performed in a blinded set-up.

### Mechanical Hyperalgesia

Before experiments, mice were exposed to the equipment without any nociceptive stimulation during 1–2 h per day for 2 days. On the third day, mice were placed in the test environment 15–20 min before testing. Baseline responses were determined three times prior to induction of priming. Mechanical hyperalgesia was measured using von Frey hairs as described (Willemen et al., 2010). The 50% paw withdrawal threshold was calculated using the up-and-down method (Chaplan et al., 1994). In short, animals were placed on a wire grid bottom through which the von Frey hairs were applied (bending force range from 0.02 to 1.4 g, starting with 0.16 g; Stoelting, Wood Dale, IL, United States). The hair force was increased or decreased according to the response. Clear paw withdrawal, shaking or licking were considered as nociceptive-like responses. Ambulation was considered an ambiguous response, and in such cases the stimulus was repeated.

### Hyperalgesic Priming

To induce hyperalgesic priming, 5 μl GDNF (2 μg/ml in saline, B&D Systems, Inc., Minneapolis, MN, United States) was intraplantarly injected into both hind paws of male and female mice (Ferrari et al., 2010). Control mice received 5 μl saline. On day 14 after GDNF priming, mice received an intraplantar injection of 2.5 μl PGE_2_ (40 μg/ml; Sigma-Aldrich) per paw. An equal volume of saline was injected as vehicle control.

### Effect of ESI-09 on GDNF-Induced Priming

ESI-09 was dissolved in ethanol (5 mg/mL) followed by dilution in corn oil (1:1) and speed vacuum to remove ethanol. To reverse priming hyperalgesia, ESI-09 (20 mg/kg) was given orally on the 13^th^ and 14^th^ day of GDNF-priming to male and female mice.

### Immunohistochemical Staining of GRK2 in DRGs After Priming

Female mice were deeply anesthetized with an overdose of sodium pentobarbital, and then transcardially perfused with PBS followed by 4% paraformaldehyde after which dorsal root ganglions (DRGs) (L3-L5 and T6-T10 from both sides) were isolated. The tissues were post-fixed, cryo-protected in sucrose in PBS, embedded in OCT compound and frozen at -80°C. Frozen sections of DRGs (10 μm) were stained with biotinylated isolectin B4 (IB4, 10 μg/ml, Vector Laboratories) and rabbit-anti-GRK2 (1:100, Santa Cruz Biotechnology) overnight at 4°C. Primary anti-GRK2 antibody blocked with a GRK2 blocking peptide (Santa Cruz Biotechnology) was used as a control. The slides were then incubated with Alexa-488-conjugated streptavidin (1:200, Invitrogen) and Alexa-594-conjugated goat-anti-rabbit antibody (1:200, Invitrogen). Sections were photographed with an EVOS fl (AMG, Inc.), and the GRK2 levels in IB4(+) and IB4(-) small-diameter DRG neurons (diameter < 23 μm) and large diameter DRG neurons (>30 μm) were analyzed with NIH ImageJ software.

### Image Analysis

For each mouse, 5∼7 images (format 16-bit) of lumbar (L3–L5) or thoracic (T6–T10) DRGs were taken for immunofluorescence quantification. Exposure times of photographs were identical for all slides. All stainings were done in parallel for DRGs from saline- and GDNF-treated mice. All neurons in each image were divided into three groups according to their diameter and IB4 staining as follows: small (diameter < 23 μm) IB4(+) neurons, small IB4(-) neurons (diameter < 23 μm) and large neurons (>30 μm). Cell bodies were outlined manually and the fluorescence intensity was quantified using NIH ImageJ software. The average background fluorescence of DRGs neurons (primary GRK2 antibody plus GRK2 blocking peptide) was subtracted and subsequently, the average GRK2 immunofluorescence intensity in IB4(+) neurons of naive mice was set at 100%. The percentage of GRK2 immunofluorescence in each cell was then calculated using:

$$\frac{\text{immunofluorescence intensity of the neurons}}{\text{The average GRK2 immunofluorescence intesity in IB4}(+)\text{ neurons of naive mice}}\times 100\%$$

### Herpes Simplex Virus (HSV)-Mediated GRK2/GFP Gene Expression

Herpes simplex virus (HSV)-GRK2/GFP was generated by using a replication-deficient HSV-vector into which we cloned GRK2 under control of the α4 promoter and as a reporter gene green fluorescent protein (GFP) under control of the α22 promoter (Zhao et al., 2004; Wang et al., 2013). As a control we used HSV-GFP containing GFP only. Female mice were inoculated intraplantarly twice with HSV-GRK2/GFP [2.5 μl of 1.4 × 10^7^ plaque forming units (pfu)/ml] at 4 and 2 days before PGE_2_ injection.

### Data Analysis

Data are expressed as mean ± SEM. For behavioral data, statistical analysis was carried out using repeated measures two-way ANOVA with time as the within subject factor followed by Bonferroni’s analysis. For the data of GRK2 immunofluorescence, statistical analysis was carried out using Student’s *t*-test between saline and GDNF-primed mice, in IB4(+), IB4(-) and large neurons respectively. A *P*-value < 0.05 was considered to be statistically significant. The study design used parallel groups and investigator blinding. The data were analyzed using GraphPad Prism 6.

## Results

### GDNF-Induced Hyperalgesic Priming

**FIGURE 1:**
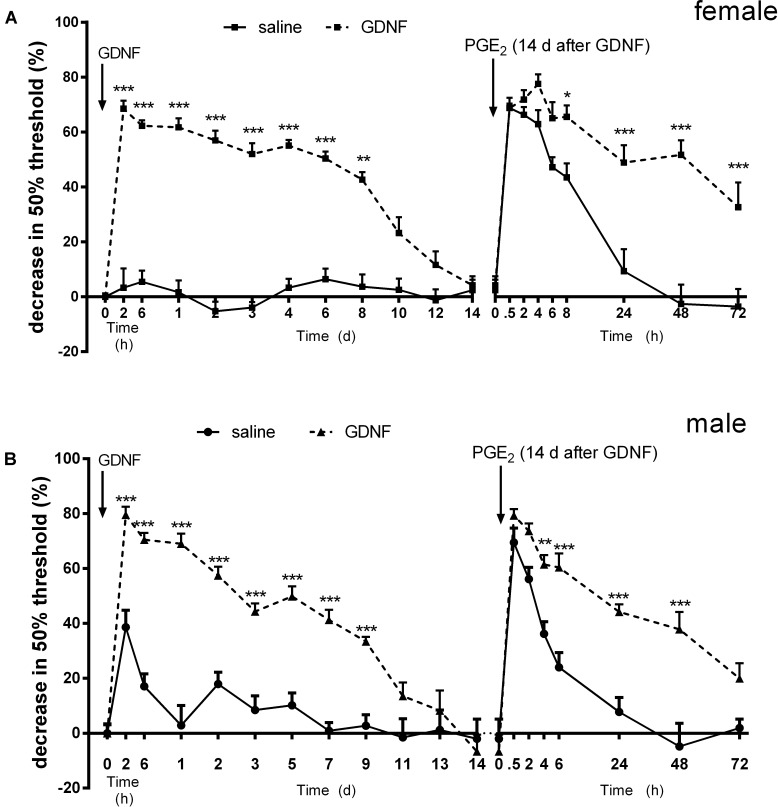
Glial cell-derived neurotrophic factor (GDNF) induces hyperalgesic priming in female and male mice. The effect of intraplantar GDNF (10 ng) or saline on mechanical sensitivity and the subsequent response to PGE_2_ in male **(A)** and female **(B)** mice. 14 days after intraplantar injection of GDNF or saline, mice received an intraplantar injection of PGE_2_ (100 ng/paw). The 50% paw withdrawal thresholds were monitored over time. Data are expressed as mean ± SEM. *n* = 8/group. **(A)**, *F* = 401.03; **(B)**, *F* = 300.74; DFn = 1, DFd = 280. Bonferroni’s multiple comparisons test: ^∗^*P* < 0.05, ^∗∗^*P* < 0.01, ^∗∗∗^*P* < 0.001.

Hyperalgesic priming was induced in male and female mice with a single intraplantar injection of GDNF (10 ng/5 μl/paw) (Ferrari et al., 2010). GDNF-induced hyperalgesia lasted ∼12 days and at day 14 after GDNF injection, the pain thresholds had returned to baseline in both sexes (**Figures 1A,B**).

When the mice subsequently received an injection of PGE_2_ at day 14 after GDNF injection, PGE_2_-induced mechanical hyperalgesia lasted more than 72 h in both sexes. This is significantly longer than the PGE_2_ hyperalgesia in saline-treated mice, which starts to decline at 8 h and completely resolves within 24 h (*P* < 0.001; **Figures 1A,B**). These data show that GDNF induces hyperalgesic priming in both male and female mice.

**FIGURE 2:**
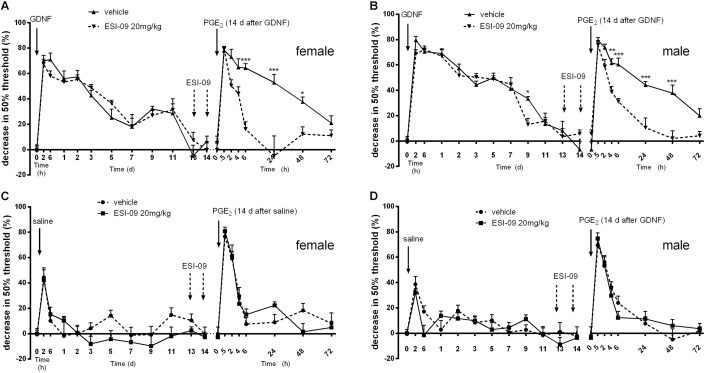
Effect of ESI-09 on GDNF-induced hyperalgesic priming. Female **(A,C)** and male **(B,D)** mice were treated with two oral doses (arrows) of ESI-09 (20 mg/kg in corn oil) or vehicle on days 13 and 14 after GDNF priming or saline. One hour after the last ESI-09 administration, the GDNF-primed mice received an intraplantar injection of PGE_2_ (100 ng/paw). The 50% paw withdrawal thresholds were monitored over time. Data are expressed as mean ± SEM. *n* = 8/group. **(A)**, *F* = 26.41; **(B)**, *F* = 33.49; **(C)**, *F* = 2.31; **(D)**, *F* = 0.58; DFn = 1, DFd = 280. Bonferroni’s multiple comparisons test: ^∗^*P* < 0.05, ^∗∗^*P* < 0.01, ^∗∗∗^*P* < 0.001.

To determine the contribution of Epac signaling to GDNF-induced priming, we used the Epac inhibitor ESI-09 (Singhmar et al., 2016). In both male and female mice, treatment with ESI-09 (20 mg/kg, orally) before PGE_2_ administration significantly reduced the duration of PGE_2_ hyperalgesia in mice primed with GDNF (**Figures 2A,B**). In contrast, ESI-09 treatment did not affect the duration and severity of PGE_2_ hyperalgesia in naive mice (**Figures 2C,D**). The data in **Figure 2** also show that ESI-09 treatment did not affect pain sensitivity of GDNF-primed mice of both genders in the absence of PGE_2_ stimulation. Together, our findings indicate that the prolongation of PGE_2_ allodynia after GDNF priming is associated with engagement of an Epac-mediated PGE_2_-signaling pathway that is not operative in control mice.

### GRK2 Levels in DRG Neurons of GDNF-Primed Mice

We next used immunofluorescence analysis to determine whether GDNF hyperalgesic priming and the engagement of Epac signaling are associated with a change in GRK2 protein level in lumbar and thoracic DRGs at 14 days after intraplantar administration of GDNF. Since the non-peptidergic IB4(+) subpopulation of DRG neurons is known to be involved in GDNF-mediated hyperalgesic priming (Joseph and Levine, 2010), we used IB4 as a marker to identify this specific subgroup of DRG neurons.

**FIGURE 3:**
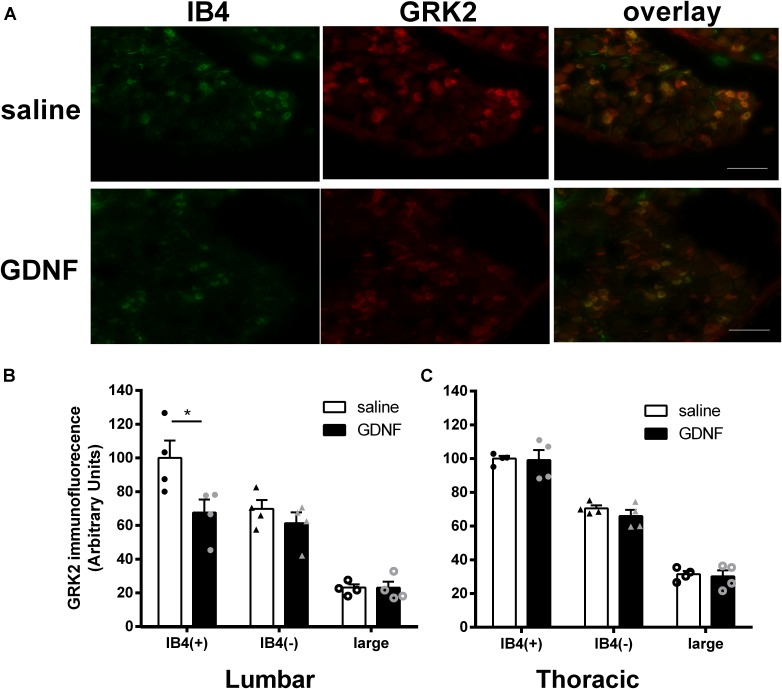
Changes in GRK2 level in DRG neurons of GDNF-primed mice. Male mice received an intraplantar injection of GDNF (10 ng). 14 days later GRK2 protein levels in IB4(+) and IB4(–) small (<23 μM) diameter neurons and in large (>30 μM) neurons from lumbar and thoracic DRGs were quantified by immunofluorescence analysis. **(A)** Representative pictures of IB4 (green) and GRK2 (red) double staining of lumbar DRGs from saline and GDNF-treated mice. Bar graphs representing average GRK2 immunofluorescence intensities in **(B)** lumbar or **(C)** thoracic DRG at 14 days after intraplantar GDNF or saline. Data are expressed as mean ± SEM of four mice per group and are based on ∼150 neurons from L3–L5 or T6–T10 DRGs per mouse. Student’s *t*-test: lumbar IB4(+), *t* = 2.520, df = 6, ^∗^*P* = 0.0453; lumbar IB4(–), *t* = 1.019, df = 6, *P* = 0.3475; lumbar large, *t* = 0.02252, df = 6, *P* = 0.9828; Thoracic IB4(+), *t* = 0.1383, df = 6, *P* = 0.8945; Thoracic IB4(–), *t* = 1.136, df = 6, *P* = 0.2994; Thoracic large, *t* = 0.3275, df = 6, *P* = 0.7544; The variances were homoscedastic tested by *F*-test. Scale bar: 100 μm.

**Figure 3A** shows the expression of GRK2 protein in DRGs as visualized by double immunofluorescence with anti-GRK2 antibody and IB4. At 14 days after intraplantar GDNF injection into the hind paws, the level of GRK2 in IB4(+) small diameter (<23 μm) neurons was reduced by ∼30% in lumbar DRGs (**Figure 3B**). We did not observe any change in GRK2 protein levels in IB4(-) small diameter neurons in lumbar DRGs after GDNF priming (**Figure 3B**). GRK2 levels in medium or large diameter (>30 μM) neurons were lower than in small diameter neurons but were not affected by GDNF priming (**Figure 3B**). In addition, we did not detect changes in the percentage of IB4 + neurons or GRK2+ neurons (data not shown).

Glial cell-derived neurotrophic factor administration into the hind paw did not change GRK2 protein levels in IB4(+) and IB4(-) small diameter neurons in thoracic DRGs (**Figure 3C**).

Our previous study showed that hyperalgesic priming had no effect on DRG morphology (Wang et al., 2011). In the present study, we also check the numbers of IB4(+), IB4(-), and large neurons in both naive and GDNF-primed mice. The results showed that the GDNF-priming also had no effect on the numbers of DRG neuron distribution, including small IB4(+), small IB4(-), and large DRG neurons (data not shown).

### Effect of GRK2 Gene Transfer on GDNF-Induced Hyperalgesic Priming

To determine whether the decreased neuronal GRK2 level in GDNF-prime animals is responsible for the prolongation of PGE_2_ hyperalgesia, we examined the effect of overexpressing GRK2 on hyperalgesic priming induced by GDNF. To overexpress GRK2 in primary sensory neurons, we used a Herpes simplex virus vector containing the GRK2 gene and green fluorescent protein (GFP) as a reporter gene (Wang et al., 2013). As a control, the same HSV vector containing GFP only was used. Our previous work has shown that two intraplantar inoculations of this HSV construct was sufficient to express the reporter GFP protein in DRG neurons, sciatic nerve fibers, and peripherin^+^ nerves in the skin (Wang et al., 2013). These findings indicate successful overexpression of the transgene in functionally relevant neurons.

**FIGURE 4:**
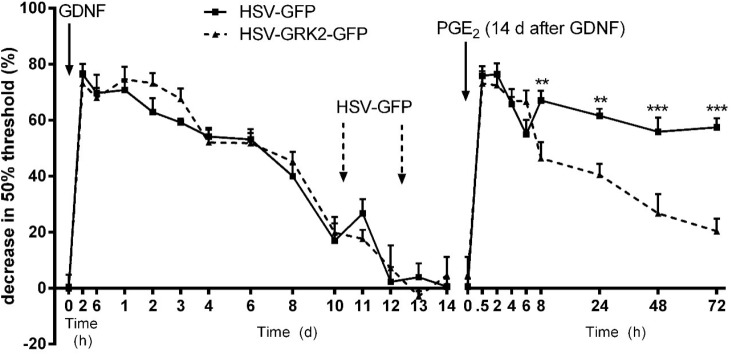
Effect of GRK2 overexpression on GDNF-induced hyperalgesic priming. GDNF-induced hyperalgesia and effect of two intraplantar injections (arrows) of HSV-GRK2-GFP or HSV-GFP (1.4 × 10^7^ pfu/mL, 2.5 μL/paw). Two days after the last HSV inoculation, the GDNF-primed mice were treated with PGE_2_ (100 ng/paw). The 50% paw withdrawal thresholds were monitored over time. Data are expressed as mean ± SEM. *n* = 6/group. *F* = 27.81; DFn = 1, DFd = 230. Bonferroni’s multiple comparisons test: ^∗∗^*P* < 0.01, ^∗∗∗^*P* < 0.001.

At day 10 and day 12 after intraplantar GDNF administration, the mice received intraplantar injections with 2.5 μl of 1.4 × 10^7^ pfu/ml HSV-GRK2/GFP or control HSV-GFP. HSV-GRK2/GFP-mediated overexpression of GRK2 in DRG neurons significantly shortened PGE_2_-induced hyperalgesia after GDNF priming (**Figure 4**) compared to mice treated with the same dose of control HSV-GFP.

## Discussion

Understanding the mechanisms underlying transition from acute to chronic pain is thought to be key to the development of novel interventions to prevent chronic pain. This knowledge is needed because once chronic pain is established, it is difficult to treat. In search for mechanisms underlying transition to chronic pain, we used hyperalgesic priming as a model of long-lasting latent hyperresponsiveness of nociceptors to an inflammatory insult. In this model, mechanical allodynia in response to a single injection of PGE_2_ is prolonged when administered after recovery from a previous inflammatory stimulus. Here, we show that GDNF-induced hyperalgesic priming decreases GRK2 in DRG neurons and leads to engagement of an Epac-mediated signaling pathway leading to prolonged PGE_2_-induced hyperalgesia. Increasing the GRK2 protein level in DRG neurons by gene-transfer using HSV-GRK2 in primed mice or inhibiting Epac1 activity by ESI-09 reversed the prolongation of PGE_2_ hyperalgesia in GDNF-primed mice.

IB4(+) DRG neurons, which are non-peptidergic nociceptors, express Ret, the receptor for GDNF (Molliver et al., 1997; Bennett et al., 1998). It has been shown that ablation of IB4(+) DRG neurons by administration of the cell-selective neurotoxin IB4-saporin eliminates both GDNF-induced hyperalgesia and GDNF-induced priming (Joseph and Levine, 2010). We now show that the activation of IB4(+) neurons through intraplantar administration of GDNF induces a persistent decrease in the level of GRK2 protein specifically in IB4(+) cells but not in IB4(-) DRG small or large diameter neurons. Notably, the decrease in GRK2 protein in IB4(+) neurons was observed at a time point at which GDNF-induced hyperalgesia had already completely resolved. This decrease in GRK2 protein level represents a neuroplastic change induced by GDNF that persists after recovery from GDNF-induced hyperalgesia. The decrease in GRK2 protein in IB4(+) DRG neurons does not change the basal sensitivity to mechanical stimulation, but prolongs mechanical allodynia induced by a second stimulus, i.e., PGE_2_ in our experiments. These observations are in line with data we obtained in mice with genetic deletion of GRK2 in Na_V_1.8-positive neurons. In these SNS-GRK2^+/-^ mice, we also showed that baseline pain sensitivity is normal, whereas PGE_2_-induced hyperalgesia is prolonged (Eijkelkamp et al., 2010b; Wang et al., 2011).

Thus, our present study offers more evidence for the mechanisms of hyperalgesic priming. According to the characteristics of hyperalgesic priming, recent studies divide the priming states into two types. Type 1 priming is IB4(+) nociceptor-dependent and effected through the PKCε signal pathway (Araldi et al., 2015, 2016a,b, 2017). Carrageenan, GDNF and the PKC agonist ΨεRACK all have been shown to induce type I priming. Hereby, our present and previous results show that GRK2 is important in the type I priming. Both carrageenan and GDNF injections decreased GRK2 levels specifically in IB4(+) nociceptors, leading to activating of an Epac1-mediated signaling pathway in response to PGE_2_. However, we showed previously that the PKCε activator ΨεRACK induced priming and engagement of Epac signaling in response to PGE_2_ without decreasing the level of GRK2. ΨεRACK priming turned out to be associated with an increase in Epac1 protein level and activity. Nevertheless, increasing GRK2 using the same viral construct used in our present study reversed the primed phenotype, suggesting that the balance between GRK2 and Epac1 controls engagement of the Epac signaling pathway. The role of GRK2 in type 2 priming, which is PKCε independent, needs to be further studied.

We previously investigated the molecular mechanism via which low GRK2 leads to engagement of Epac signaling in response to stimulation with PGE_2_ or other cAMP-inducing agents. Epac signaling in nociceptors induces persistent pain via activation of MEK/ERK and PKCε (Eijkelkamp et al., 2010b). We showed that SNS-GRK2^+/-^ mice who have a 50% reduction in nociceptor GRK2 protein, developed prolonged hyperalgesia in response to the Epac activator, 8-pCPT-2′-O-Me-cAMP (8-pCPT) when compared to WT mice. Moreover, we showed that the prolongation of PGE_2_-induced pain in GRK2^+/-^ mice was prevented by the antisense oligonucleotide mediated down regulation of Epac1 (Wang et al., 2013) while intraplantar injection of HSV-GRK2 reduced Epac1-dependent CFA-induced inflammatory mechanical hyperalgesia (Singhmar et al., 2016), demonstrating that GRK2 regulates Epac1-dependent hyperalgesia. Co-immunoprecipitation experiments showed that GRK2 binds to Epac1. Our recent studies further showed that *in vitro*, GRK2 inhibits Epac1-to-Rap1 signaling by phosphorylation of Epac1 at Ser-108 in the Disheveled/Egl-10/pleckstrin domain. This phosphorylation event inhibits agonist-induced translocation of Epac1 to the plasma membrane, thereby reducing Rap1 activation, resulting in the prevention of the transition from acute to chronic pain (Singhmar et al., 2016). The present study demonstrates that GDNF-induced priming is associated with a reduction in nociceptor GRK2 and engagement of Epac signaling as well. In both male and female GDNF-primed mice, systemic administration of Epac inhibitor ESI-09, prevented the prolonged PGE_2_ hyperalgesia, indicating Epac pathway activation in these primed mice with low nociceptor GRK2 level.

Local targeted overexpression of GRK2 in DRG neurons by intraplantar administration of HSV-GRK2 amplicons prevented the transition to persistent PGE_2_ hyperalgesia after GDNF-priming. This finding indicates that the GDNF-induced decrease in nociceptor GRK2 is causally related to the transition to chronic hyperalgesia in sensitized mice. Therefore, we propose that the decrease in nociceptor GRK2 induced by GDNF priming is a key component of the neuroplastic changes that are induced in this model as a consequence of hyperalgesic priming.

Levine and co-workers described that a transient reduction in GRK2 protein levels in the DRG as induced by intrathecal GRK2 antisense application continued to prolong PGE_2_ hyperalgesia, even after GRK2 protein levels had returned to baseline. They referred to this phenomenon ‘GRKing’ (Ferrari et al., 2012). However, we show here that restoring the decreased GRK2 levels in sensory neurons using HSV-GRK2 amplicons in mice primed with GDNF directly reversed the prolongation of PGE_2_ hyperalgesia. There are two possible explanations for the differences between Levine’s and our study. First, it is possible that HSV-mediated overexpression of GRK2 increases GRK2 levels in sensory neurons above baseline levels, thereby reversing a potential effect of transient decreases in GRK2. It is also possible that the intrathecal GRK2 antisense ODN affects GRK2 levels in cells in the spinal cord other than nociceptors in DRG, including microglia, astrocytes and spinal cord neurons, leading to the ‘GRKing’ phenotype.

Herpes simplex virus-mediated gene transfer has been suggested to be an ideal strategy for gene therapy of chronic pain in a few recent studies (Guedon et al., 2015; Thakur et al., 2016; Meidahl et al., 2017). It has a natural ability to infect predominantly sensory nerves after peripheral administration and provides long-term protein expression in a restricted appropriate region of the body (Goss et al., 2009). Indeed, we have shown that intraplantar injections of HSV-GRK2-GFP results in successful expression of the GFP reporter protein in DRG neurons, sciatic nerve fibers and the nerve ending in the footpad (Wang et al., 2013). After recovery from the transient mild hyperalgesia immediately after HSV administration, pain sensitivity returned to baseline rapidly, indicating that the viral vector does not induce persistent hyperalgesia by itself. The results of a recent phase 1 clinical trial investigating the safety of HSV-mediated gene transfer for the treatment of pain are encouraging; intradermal HSV-treatment was well-tolerated and the encephalin-encoding vector used in that study may indeed have analgesic effects (Wolfe et al., 2009; Fink et al., 2011).

G protein coupled receptor kinase 2 is known to be highly expressed in immune and nervous system and the level of GRK2 protein is affected by many diseases. For example, the expression of GRK2 was reduced in splenocytes in the rats with experimental autoimmune encephalomyelitis (EAE), an animal model of relapsing-remitting multiple sclerosis (MS). The levels of GRK2 were also decreased in immune cells in the complete Freunds adjuvant animal model of arthritis. Clinically, in peripheral blood mononuclear cells of patients with active MS or with secondary progressive MS, as well as in lymphocytes of patients with rheumatoid arthritis (RA), GRK2 level were shown to be reduced (Lombardi et al., 1999, 2001; Vroon et al., 2003, 2005). Chronic pain is known to develop in these inflammatory autoimmune diseases. But, it remains to be determined whether the low GRK2 protein level as observed in leukocytes is mirrored by a decrease in GRK2 in DRG neurons of these patients as well.

## Conclusion

The efficacy of the GDNF-primed state of sensory neurons of the DRG to induce transition to chronic pain is determined by the protein level of GRK2 or Epac1 activity in IB4 positive neurons. The primed state can be reversed by increasing the level of GRK2 or inhibiting Epac1 activity.

## Author Contributions

H-JW and NE designed the study, conducting the experiments, and drafted the manuscript. H-XG performed experiments. CH and AK supervised the study, contributed to writing, and editing the manuscript. All authors read and approved the final manuscript.

## Conflict of Interest Statement

The authors declare that the research was conducted in the absence of any commercial or financial relationships that could be construed as a potential conflict of interest.
